# The relationship between the effect of matured hop extract and physical activity on reducing body fat: re-analysis of data from a randomized, double-blind, placebo-controlled parallel group study

**DOI:** 10.1186/s12937-018-0405-3

**Published:** 2018-10-30

**Authors:** Shigeo Suzuki, Takahiro Yamazaki, Chika Takahashi, Yuji Kaneko, Yumie Morimoto-Kobayashi, Mikio Katayama

**Affiliations:** Research Laboratories for Health Science and Food Technologies, Kirin Co., Ltd. 1-13-5, Fukuura, Kanazawa-ku, Yokohama, 236-0004 Japan

**Keywords:** Matured hop extract, Matured hop bitter acids, Body fat, Obesity, Physical activity, Interaction effect

## Abstract

**Background:**

We recently reported that successive ingestion of matured hop extract (MHE), produced by oxidation of hops, results in a reduction of body fat in healthy overweight participants. A combined effect of MHE and physical activity on body fat has not been investigated. Thus, we re-analyzed data from the previous study to explore the relationship between the effect of MHE and walking as an index of physical activity.

**Methods:**

This analysis uses existing data from a randomized, double-blind, placebo-controlled parallel group study in which MHE (active) or placebo was given for 12 w to 200 healthy overweight Japanese, from May to December 2014. Correlation between the change in abdominal fat areas at 12 w and the number of steps taken per day was tested by Spearman’s correlation coefficient test. The subjects were stratified using the average number of steps per day of Japanese into walking less and walking more subgroups (WL and WM, respectively) as follows: placebo (WL, *n* = 43; WM, *n* = 44) and active (WL, *n* = 49; WM, *n* = 42). Reductions in total, visceral, and subcutaneous fat area (TFA, VFA and SFA, respectively) were evaluated. The interaction effect between ingestion (active/placebo) and walking (WL/WM) was analyzed using two-way analysis of variance (ANOVA).

**Results:**

There was a significant negative correlation between the change in VFA and daily steps taken in the active group (*r* = − 0.208, *P* = 0.048). No significant correlation in TFA or SFA. Although the interaction effect in TFA was not significant, the main effect of ingestion was significant (*P* = 0.045). In contrast, the interaction effect in VFA was suggested to be synergistic (*P* = 0.055).

**Conclusion:**

The results suggested that MHE ingestion combined with light intensity exercise would induce a greater reduction in VFA which would be beneficial for obese or overweight individuals in reducing obesity and obesity-related diseases.

**Trial registration:**

UMIN-CTR UMIN000014185 registered 6 June 2014.

**Electronic supplementary material:**

The online version of this article (10.1186/s12937-018-0405-3) contains supplementary material, which is available to authorized users.

## Introduction

Obesity is increasing at an alarming rate and it is estimated that the number of obese individuals is more than 300 million worldwide [1]. Obesity increases the risk of various diseases, such as diabetes mellitus [2, 3], cardiovascular disease [4–6] and hypertension [7, 8]. To prevent obesity-related diseases, weight loss through suppressing energy intake, promoting energy expenditure, or both is a necessity in order to avoid the use of medication. Extensive research on the development of drugs [9] and functional foods [10, 11] with the potential to prevent obesity has been reported; however, there is still insufficient clinical evidence to demonstrate they have anti-obesity effects.

Abdominal obesity is classified into two types, i.e., subcutaneous and visceral fat obesity [12]. Subcutaneous fat obesity is the accumulation of fat in the peripheral area of the abdomen; visceral fat obesity is the accumulation of fat around and within the abdominal solid organs. Visceral fat obesity is more closely associated with obesity-related diseases [13–15], suggesting it is highly important to reduce visceral fat, in particular.

In general, an energy imbalance (i.e., energy expenditure exceeding energy intake) is needed to lose weight [16]. Exercise is considered a proper way to increase energy expenditure while calorie restriction plays an important role in decreasing energy intake; a combination of these two measures is the most effective way to reduce body fat [17]. Moderate exercise (light- to mild-intensity physical activity) would be acceptable for overweight and obese individuals. Although the combination of calorie restriction and exercise is more effective than just pursuing one of these measures, there seems to be less adherence to long-term dietary restriction, resulting in weight regain. Thus, an alternative approach to calorie restriction is required to help maintain a negative energy balance.

Some food constituents are reported to decrease body fat [10, 11] and the ingestion of these dietary supplements would be easier to maintain than calorie restriction. Iso-α-acids and matured hop bitter acids (MHBA) are produced by the isomerization and oxidation of α- and β-acids in hops, respectively. Hops are the main components of beer and provide beer with flavor and bitterness [18, 19]. Iso-α-acids have been reported to have many beneficiary effects on health [20–23], and to prevent obesity in mice and humans [24, 25]. Even though iso-α-acids reported to have anti-obesity effects, they are not used in foods, except for beer, due to their sharp and strong bitter taste. On the other hand, α-acid oxides may result in agreeable bitterness than iso-α-acids [26]. Because of this, MHBA have a mild bitterness which may be preferable in a variety of foods. A clinical trial recently demonstrated that ingestion of matured hop extract (MHE), which contains MHBA as active components, significantly reduced body fat, particularly the visceral fat area (VFA) [27]. These facts indicate MHBA would be suitable for application in a variety of foods and dietary supplements.

To date, the combinatorial effects of exercise and calorie restriction have been reported to enhance effects on reduction of body fat [28–30]. However, little is known about the effect of functional foods in combination with physical activity. We hypothesize that increased amount of physical activity in combination with MHE ingestion would enhance body fat reduction. To explore this possibility, our previous clinical study was re-analyzed and investigated for the relationship between the effect of MHE ingestion and number of steps taken, which is considered an index of physical activity.

## Materials and methods

This study used existing data from a randomized, double-blind, placebo-controlled parallel group study in which MHE or a placebo was given for 12 w to 200 healthy overweight subjects in Japan, from May to December 2014. The study protocol was approved by the Institutional Review Boards in each attended institution, in accordance with the ethical standards established in the Helsinki Declaration and the ethical guidelines for epidemiological research of the Ministry of Education, Culture, Sports, Science and Technology, and the Ministry of Health, Labor and Welfare of Japan. This study was registered with the UMIN Clinical Trials Registry as UMIN000014185, and was conducted in compliance with the protocol. Written informed consent was obtained from all subjects. The details of the study were described in the previous report [27].

### Subjects

The subjects were male and female aged from 20- to 65-years old, with a body mass index (BMI) ranging from 25 to 30 kg/m^2^. The exclusion criteria, also described in the previous clinical study [27], were as follows: (1) use of oral medication affecting body fat or lipid metabolism; (2) constant use of dietary supplements or functional foods affecting body fat or lipid metabolism; (3) onset of possible allergy symptoms; (4) with a history of serious disease (e.g., diabetes, liver disease, kidney disease or heart disease), thyroid gland disease, adrenal gland disease, or other metabolic disorder; (5) constant ingestion of foods enriched with hop constituents; (6) excessive alcohol-drinking behavior; (7) under treatment or with a history of drug addiction or alcoholism; (8) unfavorable results of the given lifestyle questionnaire or blood test; (9) donation of over 200 mL of blood or blood components within the last one month prior to this study, or over 400 mL of blood or blood components within the last three months prior to this study; (10) with severe anemia; (11) participation in a clinical study within the last one month prior to this study or possible participation in another clinical study; (12) possible pregnancy, pregnancy or lactating; (13) any other reason for ineligibility as judged by the site investigator.

### Test beverages

We prepared 350 mL of test beverages with or without MHE (as 35 mg MHBA) for the active or placebo beverage, respectively. The nutritional composition of the beverages is shown in Additional file 1: Table S1. No discernible difference in the appearance and taste of the two beverages were observed by a controller, who was an allocation officer. A record of whether a drink was the active or the placebo was blinded to all personnel, except the controller, until the end of the experiment.

### Study design

A randomized, double-blind, placebo-controlled parallel group study was conducted over 18 weeks and consisted of a pre-ingestion period (two weeks, − 2 to 0 w), test beverage ingestion period (12 weeks, 0 to 12 w), and follow-up period without test beverage ingestion (4 weeks, 12 to 16 w). Subjects were screened for eligibility over the two weeks prior to the ingestion period. The controller randomly assigned the subjects in a ratio of 1:1 into two groups with random numbers. The assignment list was stored in a sealed container and the subjects, all investigators and study personnel, except for the controller, remained blinded over the course of the study.

During the ingestion period, each subject took a test beverage once-daily. The time of test beverage ingestion was not limited except on test days, when subjects ingested them after the test was completed. Throughout the study, the subjects were instructed to avoid overdrinking and to continue their usual eating, exercising, sleeping, smoking and drinking habits. In addition, the subjects were prohibited from using oral medications, dietary supplements, functional foods which affect body fat or lipid metabolism, and foods enriched with hop constituents. On the day before a test, subjects were prohibited from drinking alcohol and had to finish their evening meal by 22:00, after which, eating and drinking (except for water) was prohibited until the completion of the test. On the test day, smoking was prohibited until the test was completed. Discontinuance criteria of the study for the subjects were as follows: risk of the subject′s safety; difficulty of continuation of the study due to a serious adverse event or accident; continuous or serious non-compliance with the protocol by the subjects; pregnancy; any other reason for discontinuation as judged by the site investigators.

The subjects visited the hospitals at 0, 4, 8, 12 and 16 w for the following tests: interview, measurement of anthropometric and circulatory parameters, blood and urine sampling. CT scanning was performed at 0, 8, 12 and 16 w. A lifestyle questionnaire was answered by all subjects, and a pregnancy test was taken by the female subjects at the screening test. The methods for the measurements were as described previously [27]. In the previous study, the primary endpoints were the abdominal fat areas; the secondary endpoints were body weight, BMI, body fat ratio, waist circumference, hip circumference and waist/hip ratio. In this analysis, we focused on the primary endpoints of total fat area (TFA), subcutaneous fat area (SFA) and VFA since the purpose was to further evaluate the previously confirmed effects in relation to physical activity [27].

### Stratification

In this report, the subjects in the placebo and active groups were stratified by the number of steps taken in a day, which is an index of physical activity. According to the survey of the Ministry of Health, Labour and Welfare in Japan, the average number of steps a day for Japanese men and women, aged from 20- to 64-years old, are 7,970 and 6,991, respectively. The subjects in both groups were stratified by the average numbers into two subgroups, i.e., walking less and walking more (WL and WM, respectively) as follows: placebo (WL, *n* = 43; WM, *n* = 44) and active (WL, *n* = 49; WM, *n* = 42). The average number almost equally split the placebo and active groups. The flow scheme from the enrollment of subjects and the stratification is summarized in Additional file 1: Figure. S1. Background of the subjects after the stratification is shown in Additional file 1.

### Statistical analyses

Data are expressed as the means ± standard error of the mean (SEM). Student′s *t*-test was used to evaluate the significant difference of baseline characteristics and the number of steps taken between the subgroups. The significant differences in dietary composition between the subgroups during the test-beverage ingestion period (0–12 w) were analyzed by repeated one-way analysis of variance (ANOVA), and those within subgroups (0 w vs. 4, 8, and 12 w) were analyzed by one-way ANOVA followed by Dunnett’s test. Correlation between the changes in the abdominal fat areas at 12 w and daily steps taken, and between the change in VFA at 12 w and the initial (at 0 w) of BMI were tested by Spearman’s correlation coefficient test. Two-way ANOVA was conducted to assess the main effect of ingestion (placebo/active) and walking (WL/WM) and the interaction effect between the two factors. *P* values of less than 0.05 were considered to be significant. Student’s *t*-test was performed using Microsoft Excel 2010 (Microsoft, Redmond, WA). The other statistical analyses were performed using PASW statistics 18 (IBM, Armonk, NY).

## Results

### Correlation between the number of steps and the reduction in abdominal fat area

**Fig. 1:**
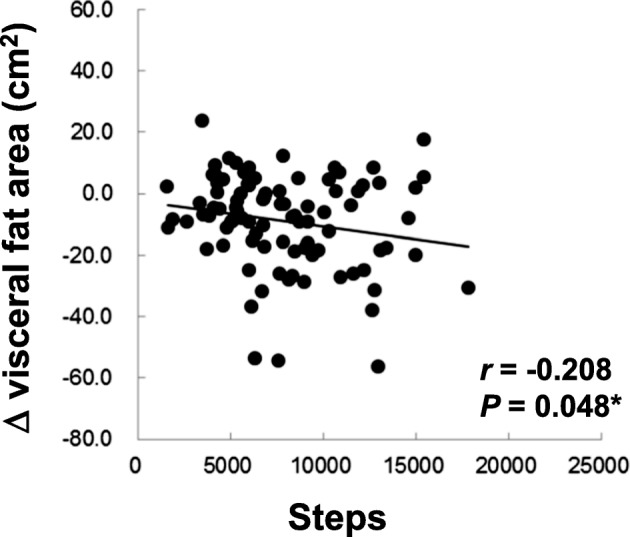
Correlation between the reduction in visceral fat area and number of steps taken per day in active group. Change in visceral fat area in the active group (*n* = 91) is illustrated. Data are calculated as the degrees of change from the initial values at 0 w (Δ). Correlation was evaluated by Spearman’s correlation coefficient test. *r*: correlation coefficient

In the previous study, it was demonstrated that ingestion of MHE significantly reduced the abdominal fat area when compared with the placebo group [27]. We hypothesized that an amount of daily physical activity would affect the effect of MHE. The relationship between the degree of change in the abdominal fat areas from 0 to 12 w and the number of steps taken a day was analyzed by Spearman’s correlation coefficient test. There was no significant correlation between the number of steps taken and reduction in TFA (*r* = − 0.048, *P* = 0.659), VFA (*r* = 0.078, *P* = 0.472) and SFA (*r* = − 0.087, *P* = 0.423) in the placebo group (Additional file 1: Figure S2D, S2E and S2F). In contrast, in the active group, there was a significant negative correlation between the number of steps taken and reduction in VFA (*r* = − 0.208, *P* = 0.048) (Fig. 1 and Additional file 1: Figure S2B). No significant correlations were not observed for TFA (*r* = − 0.089, *P* = 0.402) and SFA (*r* = − 0.009, *P* = 0.936) (Additional file 1: Figure S2A and S2C). The results suggested that the effect of MHE ingestion on reduction in VFA depended on the amount of physical activity.

### The interaction effect between MHE ingestion and walking

**Fig. 2:**
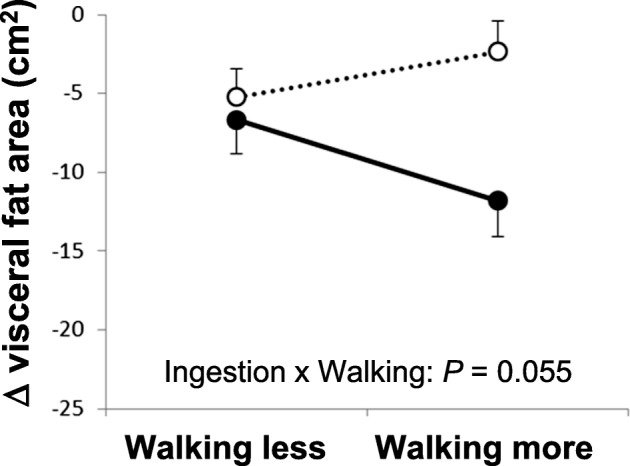
Interaction plot between the ingestion and walking effects resulting from two-way ANOVA. The degrees of change from the initial values at 0 w (Δ) were calculated for visceral fat area (VFA) of the active (solid circle and line) and placebo (open circle and dotted line) groups. The data were stratified according to the average number of steps per day taken by Japanese adults aged from 20- to 64-years old (men, 7,970 steps; women, 6,991 steps). Data are expressed as means ± SEM. For the VFA, the interaction effect (ingestion x walking) was near significant (*P* = 0.055), and the main effect of ingestion was significant (*P* = 0.010) and that of walking were not significant (*P* = 0.587)

For an in-depth analysis of the relationship between MHE ingestion and physical activity, we stratified the subjects into two subgroups by the average number of steps taken per day by Japanese adults aged from 20- to 64-years old: WL and WM. The procedure from the enrollment of subjects to the stratification is shown in Additional file 1: Figure S1. After stratification, it was considered the baseline characteristics and daily food intake did not influence the results of the re-analyzed study (Additional file 1). To investigate the relationship between MHE ingestion and walking, we analyzed the ingestion x walking interaction effect on the degree of change in the abdominal fat areas by two-way ANOVA. For the TFA, the interaction effect was not significant (*P* = 0.706); the main effect of ingestion was significant (*P* = 0.045) but that of walking was not significant (*P* = 0.443) (Additional file 1: Figure S3A). For the SFA, the interaction effect (*P* = 0.329), the main effect of ingestion (*P* = 0.376) and walking (*P* = 0.487) were not significant (Additional file 1: Figure S3C). Contrary to this, for the VFA, the main effect of ingestion was significant (*P* = 0.010) while that of walking was not significant (*P* = 0.587), and the interaction effect was near significant (*P* = 0.055) (Fig. 2 and Additional file 1: Figure S3B). In addition, the interaction effect was considered to be synergistic, according to the graph pattern (Fig. 2).

## Discussion

In our previous clinical study, the ingestion of MHE resulted in a significant decrease in TFA and VFA at 12 w and there were no adverse events related to MHE ingestion [27], as well as the safety of MHE has also been proven in a preclinical study [31]. To further investigate the effect of MHE, the study was re-analyzed to explore whether increasing daily physical activity affects the reduction in fat areas by MHE ingestion. We investigated the correlation between the change in abdominal fat areas and walking. The existing data for the degree of changes in TFA, VFA and SFA were stratified by the average number of steps taken. After stratification, the baseline characteristics and daily food intake were not associated with the results of the re-analyzed study. The interaction effect between the group and walking was also analyzed by two-way ANOVA.

We found a negative correlation between the reduction in VFA and walking (Fig. 1). In addition, a two-way ANOVA suggested that the ingestion x walking interaction effect would be synergistic in VFA (Fig. 2). Although the interaction effect was near significant (*P* = 0.055), the significant, negative correlation (Fig. 1) indicated that the higher number of steps lead to greater VFA reduction, supporting the result of the two-way ANOVA. Hence, we considered the interaction as valid. In contrast, the main effect of ingestion was significant in TFA, showing that MHE ingestion was effective on TFA independent of walking (Additional file 1: Figure S3A). These results indicate that MHE ingestion in combination with physical activity, like walking, would be more beneficial to losing VFA than MHE ingestion alone.

A reduction in visceral fat leads to a decrease in risk factors for obesity-related diseases [13–15]. Visceral adiposity is thought to be an important component of metabolic syndrome in Japan [32], for example, a reduction in visceral fat, attained by education intervention study, improved hypoadiponectinemia and cardiovascular risk factors [33, 34]; A weight loss program which included lifestyle modification and adjuvant appetite suppressant is suggested to have a greater effect on visceral fat reduction and, hence, on metabolic syndrome [35]; Visceral adipose tissue is suggested to be the most important factor in insulin resistance, and daily walking is effective on visceral fat reduction [36]. These facts indicate that visceral fat reduction has clinical significance. Our findings suggested that daily walking could enhance the reduction in VFA by MHE ingestion. Consequently, the combination would be more beneficial to obese or overweight individuals in the prevention of cardiovascular disease and diabetes mellitus.

The question arises as to why the combination of MHE ingestion and walking reduced a greater amount of VFA. Physical activity, such as walking, has been reported to reduce VFA [37]. Walking in daily life has 2.5–3.0 metabolic equivalents (METs), which stand for intensity of physical activity, and is defined as “light-intensity activity” [38]. It has been reported that obese or overweight individuals need moderate-intensity activity (METs: 3.0–5.9) between 150 and 250 min/week to lose weight [39]. In this sub-analysis, the differences in time (or energy expenditure) of daily physical activity between WM and ML were 412 min/week (1,236 kcal/week) and 321 min/week (963 kcal/week) in the active and placebo groups, respectively (Additional file 1). It appears that WM expended enough energy to reduce body fat even though the intensity was light. However, our previous study requested that subjects keep their exercise habits, rather than impose new exercise routines [27], so the energy expenditure was not derived from extra exercise.

The difference in energy expenditure between WL and WM was not attributed to the improved effect of MHE ingestion, suggesting there may be another reason why VFA would be synergistically reduced with walking in the active group. It has been demonstrated that MHBA activates the sympathetic nerve system, leading to thermogenesis in brown adipose tissue (BAT) [40]. Epithelial cells of the gastrointestinal tract have receptors which respond to various tasting components, including bitterness, and leads to the activation of the sympathetic nerve system [41]. We recently demonstrated that oral administration of MHBA induced secretion of a gastrointestinal hormone, cholecystokinin, and activated the sympathetic nerve system of BAT, inducing thermogenesis [42]. Thermogenesis could cause additional energy expenditure, in addition to the energy expended by physical activity. Stimulation of gastrointestinal receptors by MHBA would contribute to the mechanism of sympathetic nerve activation, hence, MHE ingestion is suggested to induce additional but cryptic expenditure of body fat compared to physical activity alone. In addition to thermogenesis via gastrointestinal receptors, it is known that exercise stimulates the sympathetic nerve system which then activates thermogenesis in BAT [43, 44]. Because both MHE ingestion and exercise influence thermogenesis, the two factors might affect each other and elicit a synergistic effect, leading to the enhanced reduction of VFA. Further investigation is required to elucidate the underlying mechanism of the combined effect. High-intensity exercise is difficult to achieve for overweight or obese individuals [16]. Therefore, MHE ingestion in combination with light-intensity physical activity would be acceptable for obese or overweight individuals and would be beneficial in preventing obesity related diseases.

## Conclusion

Our study showed a relationship between number of steps per day and VFA reduction in the group which ingested MHE. The re-analysis suggests that light-intensity exercise, such as walking, would promote the effect of MHE on VFA reduction. As this study is a sub-analysis of a previous analysis, further clinical studies are required to confirm our new findings. We intend to conduct an additional clinical study in combination with physical activity. The combination of MHE ingestion with light-intensity exercise could provide obese or overweight individuals with a new approach to prevent obesity and obesity-related diseases.

## Additional file

Additional file 1: **Figure S1.** Flow diagram of the progress stages of the study. **Figure S2.** Correlation between the reduction in abdominal fat area and number of steps taken per day. **Figure S3.** Interaction plot between the ingestion and walking effects resulting from two-way ANOVA. **Figure S4.** Correlation between the reduction in visceral fat area and initial values of BMI.** Table S1.** Nutritional composition of test beverages (per 100 mL). **Table S2.** Number of steps taken per day after stratification. **Table S3.** Baseline characteristics of the subjects. **Table S4.** Dietary composition. (DOCX 370 kb)

## Abbreviations

BAT: Brown adipose tissue
BMI: Body mass index
METs: Metabolic equivalences
MHBA: Matured hop bitter acids
MHE: Matured hop extract
SFA: Subcutaneous fat area
TFA: Total fat area
VFA: Visceral fat area
WL: Walking less
WM: Walking more
