# Supplementary material for: The relationship between the effect of matured hop extract and physical activity on reducing body fat: re-analysis of data from a randomized, double-blind, placebo-controlled parallel group study
Source: Nutr J. 2018 Oct 30;17:98. doi: 10.1186/s12937-018-0405-3 (PMC6208082; doi:10.1186/s12937-018-0405-3)
Supplement: Supplementary file 1 — Figure S1. Flow diagram of the progress stages of the study. Figure S2. Correlation between the reduction in abdominal fat area and number of steps taken per day. Figure S3. Interaction plot between the ingestion and walking effects resulting from two-way ANOVA. Figure S4. Correlation between the reduction in visceral fat area and initial values of BMI. Table S1. Nutritional composition of test beverages (per 100 mL). Table S2. Number of steps taken per day after stratification. Table S3. Baseline characteristics of the subjects. Table S4. Dietary composition. (DOCX 370 kb) [file 12937_2018_405_MOESM1_ESM.docx]

**Supplemental materials**

Title: The relationship between the effect of matured hop extract and physical activity on reducing body fat: re-analysis of data from a randomized, double-blind, placebo-controlled parallel group study.

**Background of the subjects**

For an in-depth analysis of the relationship between MHE ingestion and physical activity, we stratified the subjects into two subgroups by the average number of steps taken per day by Japanese adults aged from 20- to 64-years old: WL and WM. The procedure from the enrollment of subjects to the stratification is shown in Fig. S1. After stratification, the average number of steps in WL and WM were as follows: in the active group, 5,224.1 ± 220.3 and 11,063.3 ± 390.3 steps/day, respectively; in the placebo group, 5,916.0 ± 171.8 and 10,477.9 ± 419.0 steps/day, respectively (Table S2). There was significant difference between the subgroups in the active and placebo group (*P*< 0.001). According to the American College of Sports Medicine, the average number of steps can be converted into walking time (or energy expenditure) of WL and WM as follows: 52.2 ± 2.2 and 111 ± 3.9 min/day (156 ± 6.6 and 333 ± 11.7 kcal/day), respectively, in the active group; 59.2 ± 1.7 and 105 ± 4.2 min/day (178 ± 5.1 and 315 ± 12.6 kcal/day), respectively, in the placebo group. The WM took excessive physical activity by 412 min/week (1,236 kcal/week) in the active group and 321 min/week (963 kcal/week) in the placebo group, on average, when compared to the WL.

The baseline of the subjects in each subgroup is shown in Table S3. No significant difference was found in any parameter between WL and WM of the active and placebo groups, except for BMI and waist circumference. The initial BMI in WM (27.2 ± 1.1 kg/m^2^) was significantly lower than that in WL (27.9 ± 1.2 kg/m^2^) in the active group (*P* < 0.01). To confirm that the significant differences in the baselines were not associated with the results of this sub-analysis, the relationship between the initial BMI and the reduction of VFA at 12 w was evaluated by Spearman’s correlation coefficient test (Fig. S2): no correlation was observed in the active group (*r*= 0.022, *P*= 0.836) whereas there was negative but not significant correlation in the placebo group (*r*= -0.203, *P*= 0.059). These results suggested the lower initial BMI did not affect the decrease in VFA. The initial waist circumference in WM (91.7 ± 4.4 cm) was significantly lower than that in WL (94.7 ± 4.8 cm) in the placebo group (*P* < 0.001). The effect of the initial difference in waist circumference was thought to be negligible because the significant difference was not observed in the active group. In addition, dietary compositions were compared between WL and WM in the active and placebo group (Table S4). There was no significant difference between nor within the subgroups in any parameter throughout the study.

Accordingly, the baseline characteristics and daily food intake did not influence the results of the re-analyzed study.

**Supplemental Figures**


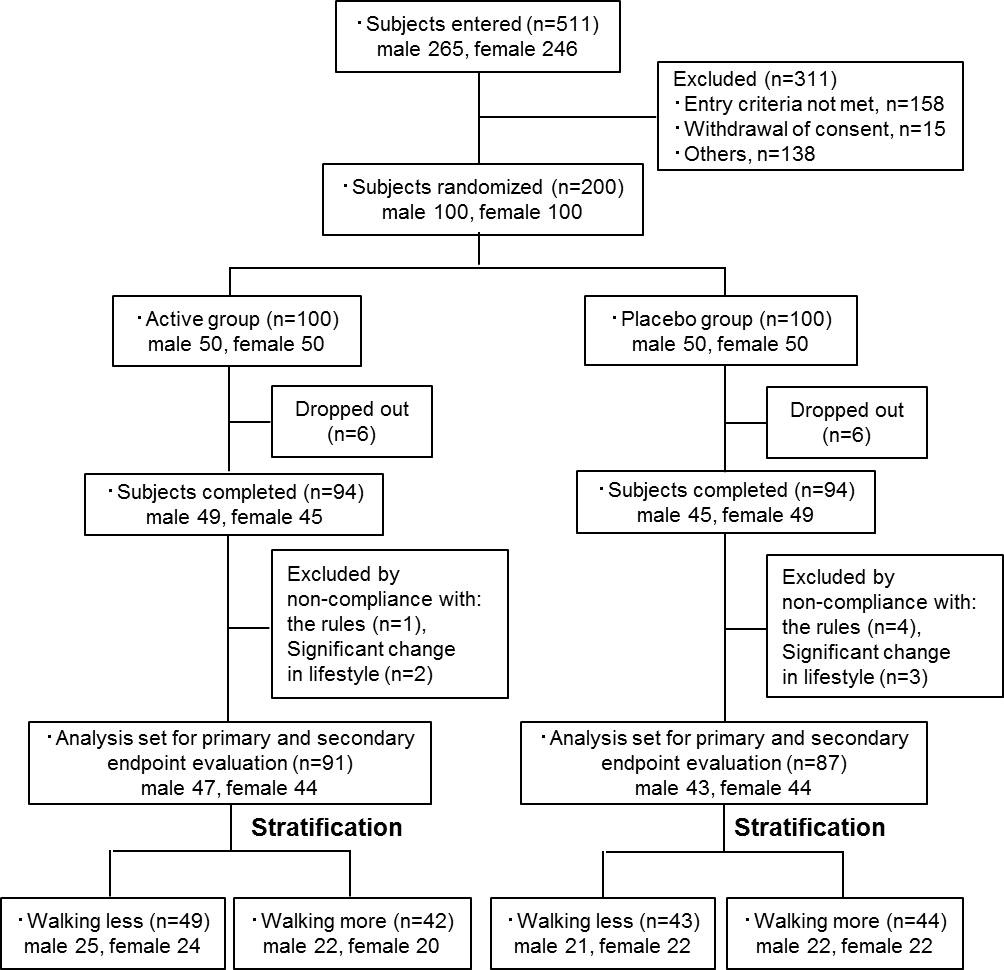


**Figure S1** Flow diagram of the progress stages of the study


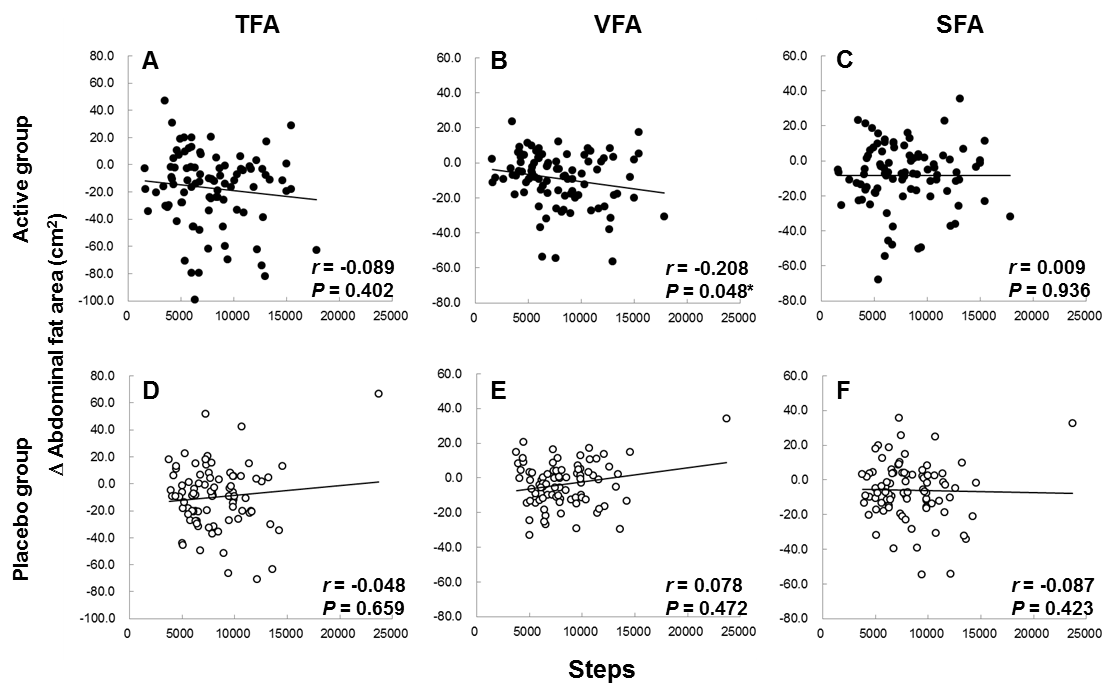


**Figure S2** Correlation between the reduction in abdominal fat area and number of steps taken per day.

Changes in total fat area (TFA, A and D), visceral fat area (VFA, B and E), and subcutaneous fat area (SFA, C and F) in the active (n=91) and placebo (n=87) groups. (A, B and C) Active group; (D, E and F) Placebo group. Data are calculated as the degrees of change from the initial values at 0 w (Δ). Correlation was evaluated by Spearman’s correlation coefficient test. r: correlation coefficient.


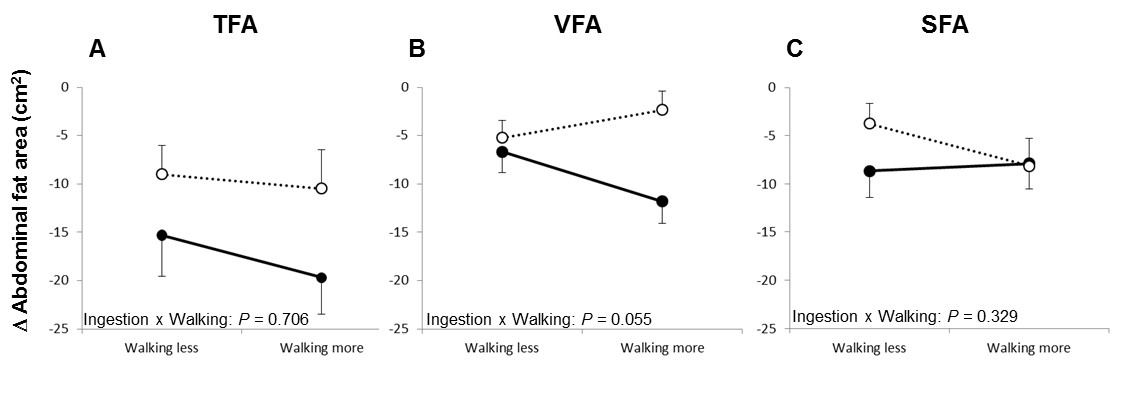


**Figure S3** Interaction plot between the ingestion and walking effects resulting from two-way ANOVA.

The degrees of change from the initial values at 0 w (Δ) were calculated for total fat area (TFA, A), visceral fat area (VFA, B), and subcutaneous fat area (SFA, C) of the active (solid circle and line) and placebo (open circle and dotted line) groups. The data were stratified according to the average number of steps per day taken by Japanese adults aged from 20- to 64-years old (men, 7,970 steps; women, 6,991 steps). Data are expressed as means ± SEM. For the TFA, the interaction effect (ingestion x walking) was not significant (*P* = 0.706); however, the main effects of ingestion was significant (*P* = 0.045) and that of walking was not significant (*P* = 0.443). For the VFA, the interaction effect (ingestion x walking) was near significant (*P* = 0.055), and the main effect of ingestion was significant (*P* = 0.010) and that of walking were not significant (*P* = 0.587). For the SFA, the interaction effect (ingestion x walking) was not significant (*P* = 0.329), and the main effects of ingestion and walking were not significant (*P* = 0.376 and *P* = 0.487, respectively).


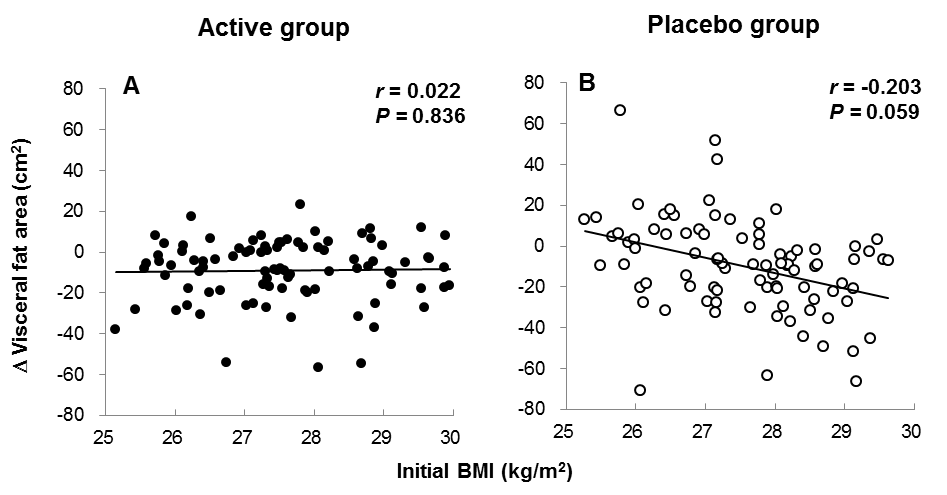


**Figure S4** Correlation between the reduction in visceral fat area and initial values of BMI

(A) Active group (n=91); (B) Placebo group (n=87). Data were calculated as the degrees of change from the initial values at 0 w (Δ). Correlation was evaluated by Spearman’s correlation coefficient test. *r*: Correlation coefficient.

**Supplemental Tables**

**Table S1** Nutritional composition of test beverages (per 100 mL)


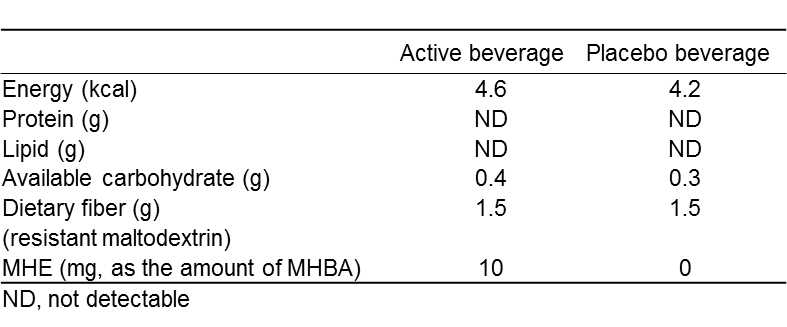
**Table S2** Number of steps taken per day after stratification


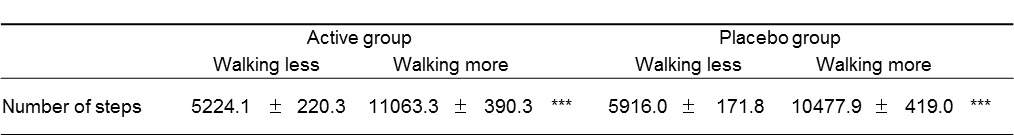


Data are shown as means ± SEM. There were significant differences between the subgroups (walking less vs. more) in the active and placebo group using Student’s *t*-test (***, *P* < 0.001).

**Table S3** Baseline characteristics of the subjects


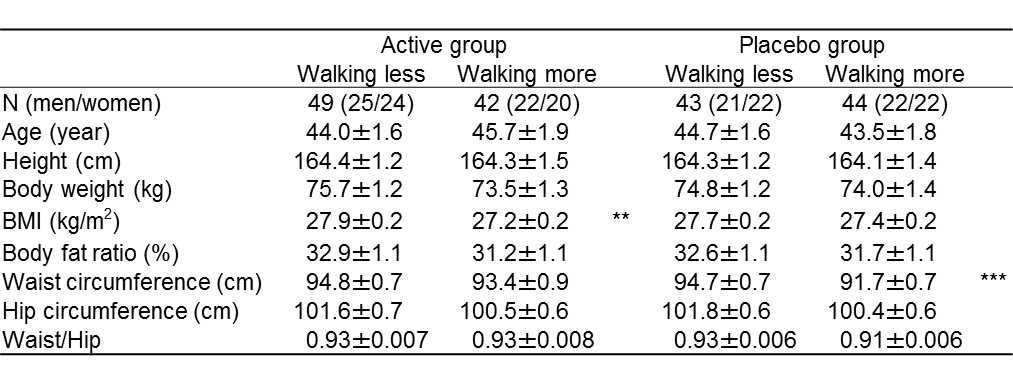


Data are expressed as means ± SEM. There were significant differences between the subgroups (walking less vs. more) in the active and placebo group using Student’s *t*-test (**, *P* < 0.01; ***, *P* < 0.001).

**Table S4** Dietary composition


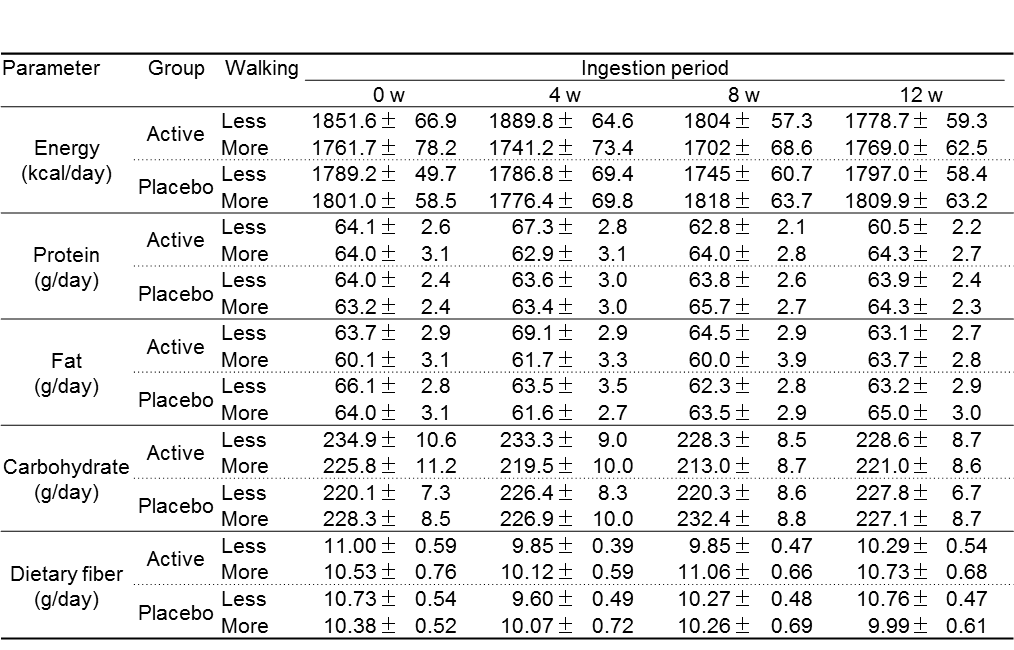


Data are shown as means ± SEM. No significant differences were observed in any parameter between the two subgroups (walking less vs. more) in the active or placebo group using repeated one-way ANOVA and within the subgroup using one-way ANOVA followed by Dunnett’s test (0 w vs. 4, 8, 12 or 16 w).
